# *In Vitro* Protective Effect of Phikud Navakot Extraction on Erythrocyte

**DOI:** 10.1155/2016/1961327

**Published:** 2016-11-28

**Authors:** Kanchana Kengkoom, Sumate Ampawong

**Affiliations:** ^1^Academic Services Office, National Laboratory Animal Center, Mahidol University, 999 Salaya, Puttamonthon, Nakorn Pathom 73170, Thailand; ^2^Department of Tropical Pathology, Faculty of Tropical Medicine, Mahidol University, 420/6 Ratchawithi Road, Ratchathewi, Bangkok 10400, Thailand

## Abstract

Phikud Navakot (PN), Thai herbal remedy in National List of Essential Medicines, has been claimed to reduce many cardiovascular symptoms especially dizziness and fainting. Apart from blood supply, erythrocyte morphology, in both shape and size, is one of the main consideration factors in cardiovascular diseases and may be affected by vascular oxidative stress. However, little is known about antioxidative property of PN on erythrocyte to preserve red blood cell integrity. In this study, 1,000 *μ*M hydrogen peroxide-induced oxidative stress was conducted on sheep erythrocyte. Three doses of PN (1, 0.5, and 0.25 mg/mL) and 10 *μ*M of ascorbic acid were compared. The released hemoglobin absorbance was measured to demonstrate hemolysis. Electron microscopic and immunohistochemical studies were also performed to characterize dysmorphic erythrocyte and osmotic ability in relation to aquaporin- (AQP-) 1 expression, respectively. The results revealed that all doses of PN and ascorbic acid decreased the severity of dysmorphic erythrocyte, particularly echinocyte, acanthocyte, knizocyte, codocyte, clumping, and other malformations. However, the most effective was 0.5 mg/mL PN dosage. In addition, hydrostatic pressure may be increased in dysmorphic erythrocyte in association with AQP-1 upregulation. Our results demonstrated that PN composes antioxidative effect to maintain the integrity and osmotic ability on sheep erythrocyte.

## 1. Introduction

Erythrocyte is an important target reactive site of oxidative stress due to its membrane composing of high concentration of polyunsaturated fatty acid and hemoglobin molecule, the potent accelerators of reactive O_2_ species (ROS) [1]. Although erythrocyte has its own pathway, both enzymatic and nonenzymatic, acting as an antioxidative mechanism to protect and counteract those generated ROS [2], the imbalance production of ROS and antioxidant on erythrocyte causes a number of cardiovascular diseases such as acute coronary syndrome [3], hypercoagulation [4], endothelial cell dysfunction [5], and diabetic induced cardiovascular defects [6]. Several studies have suggested that fruit, vegetables, and cereals together with herbs may alleviate or prevent oxidative stress on erythrocyte [7–10]. Phikud Navakot (PN), major ingredient of Yahom Navakot with powder form for drink maker, is a combination of nine Thai herbal plant species,* Anacyclus pyrethrum*,* Angelica dahurica*,* Angelica sinensis*,* Atractylodes lancea*,* Artemisia annua*,* Ligusticum sinense*,* Picrorhiza kurroa*,* Saussurea lappa*, and* Terminalia chebula*. It is also listed in the National Public Health Ministry of Thailand's list of herbal medical products. Our previous studies demonstrated that PN has several advantage effects to cardiovascular system, for instance, vasorelaxation [11], blood flow enhancement [12], and induction of glucose catabolism [12]. Recently, we found that PN ameliorates endothelial stress, attenuates oxidative stress induced by apoptosis, and prevents platelet aggregations [13]. However, high dose oral administration and prolong usage are contraindication in association with mesangiolysis [14]. Unfortunately, there is no report relevant to the protective role of PN extraction against oxidative damage on red blood cell. Consequently, the aim of the present study is to characterize an antioxidative property of PN on erythrocyte, performed by an* in vitro* model, hydrogen peroxide-induced oxidative stress on sheep erythrocyte. Spectroscopic, electron microscopic, and immunohistochemical studies were used as tools to demonstrate antioxidative effect of PN in relation to ultrastructural changes and osmotic ability.

## 2. Materials and Methods

### 2.1. Herbal Materials

PN extraction was kindly provided and prepared by Associate Professor Dr. Uthai Sotanaphun, Department of Pharmacognosy, Faculty of Pharmacy, Silpakorn University, Nakorn Pathom, Thailand. The herbs in PN remedy were mixed, ground, and then immersed in 80% ethyl alcohol for overnight. The mixture was boiled and filtrated to remove the residual as twice. Finally, to remove the trace solvent, the aqueous extraction was spray-dried.

### 2.2. Sheep Blood Collection and Erythrocyte Isolation

Alsevers sheep blood was obtained from Salaya Animal Hospital. Sheep blood derived specimens conducted in this experiment were approved by National Laboratory Animal Center-Animal Care and Use Committee, Mahidol University (approval number RA2011.03). Erythrocyte isolation was performed as described in Alanazi, 2010, [15] with slight modifications. Alsevers blood was centrifuged for 5 min at 1,500 rpm at 4°C. The plasma and buffy coat were aspirated and discarded. The erythrocyte pellet was washed three times in cold 0.9% normal saline solution (NSS), pH 7.4, with centrifugation for 5 min at 1,500 rpm at 4°C. The isolated erythrocyte was kept cold and performed to the experiment within 6 hr. The centrifuge was kindly provided by Associate Professor Dr. Yaowapa Maneerat, Department of Tropical Pathology, Faculty of Tropical Medicine, Mahidol University.

### 2.3. Hemolysis

Hydrogen peroxide (H_2_O_2_) was used as free radical initiator [16] to determine antihemolytic activity among three doses of PN compared to ascorbic acid and nontreatment group as positive control. The concentration of 1,000 *μ*M H_2_O_2_ was selected to induce an oxidative reaction on intact sheep erythrocyte (chosen from dose titration result). The reaction was performed by the combination of PN, H_2_O_2_, and erythrocyte. The 50 *μ*L of PN (at each concentration of 1.0, 0.5, and 0.25 mg/mL) or 10 *μ*M ascorbic acid was combined with 50 *μ*L of isolated erythrocyte, 50 *μ*L of 1,000 *μ*M H_2_O_2_, and 50 *μ*L of 0.9% NSS. The mixtures were gentle mixed and incubated in 37°C for 1 hr. The erythrocyte suspension was centrifuged for 5 min at 1,500 rpm at 4°C. The released hemoglobin absorbance was then measured in the supernatant at 540 nm. The level of hemolysis was determined by the intensity of released heme from damaged red blood cell characterized by its darkened color or positive OD absorptivity. The erythrocyte pellet was processed for ultrastructural and immunohistochemical studies.

### 2.4. Scanning Electron Microscopy (SEM)

The erythrocyte pellet was primary fixed with 2.5% glutaraldehyde in 0.1 M sucrose phosphate buffer, pH 7.4, and was washed 3 times with 0.1 M sucrose phosphate buffer for 10 min each. Fixed erythrocyte was smeared on a glass cover slip and air-dried for overnight. The cover slip was mounted on the aluminum stub using double-sided carbon tape and coated with gold film to 20 nm thickness using the sputter coater (EMITECH K550, UK). The erythrocyte was examined under the scanning electron microscope (JEOL JSM-6610LV, Japan) with 15 kV acceleration voltages. Ten fields of erythrocytic smear per group of the experiment were randomly examined and captured at 3,000x magnification. Each type of dysmorphic erythrocyte described in several reports [15, 17–20] was identified and counted as percentage/field.

### 2.5. Immunohistochemistry

Polyclonal rabbit anti-aquaporin- (AQP-) 1 (Millipore, USA, AB3272-200UL) was used to detect osmotic ability on erythrocytic membrane. The fixed erythrocyte pellet was smeared on precoated immunohistochemistry slides. The slides were air-dried for overnight and again fixed in absolute methanol for 3 min. The erythrocyte was unmasked for the AQP-1 antigen by heat-induced antigen retrieval using microwave in citrate buffer (pH 6). The erythrocyte was blocked from peroxidase reaction with 3% v/v hydrogen peroxide in methanol for 10 min after the slides were cooled. The erythrocyte was washed with 0.2% v/v Tween in phosphate buffered saline (PBS) 3 times, for 5 min each and blocked with protein block serum free (Dako, Denmark, X0909) for 10 min. The erythrocyte was incubated with 1 : 100 of AQP1 in PBS with 1% v/v normal goat serum (NGS, Vector, USA, S1000) at 4°C for overnight. Then, the erythrocyte was washed and incubated for 30 min with labeled polymer HRP anti-mouse/rabbit EnVision kit (Dako, Denmark, K5007) and visualized with diaminobenzidine (DAB, Dako, Denmark, K3468). The erythrocyte was then counterstained with hematoxylin and mounted by DPX.

Ten fields of erythrocytic smear per group were randomly examined and captured at 1,000x magnification. Immunohistochemical expression of AQP-1 was analyzed using ImageJ, NIH®, [21] briefly; the color images were converted to 8 bits and adjusted threshold to locate the area of expression. The positive reaction was measured as percentage area of expression/field. The intensity of AQP-1 expression was also simultaneously scored with 4 grades: 0 (no expression), 1 (mild expression), 2 (moderate expression), and 3 (strong expression). *H*-score, 0–300, was calculated by multiplication of the percentage area of expression/field and intensity score.

### 2.6. Statistical Analysis

Quantitative results were expressed as mean ± standard error of mean. Data were statistically analyzed with GraphPad PRISM® statistical software version 6.05. Nonparametric unpaired *t*-test was used to differentiate the difference between groups at 95% CI.

## 3. Results and Discussion

### 3.1. Hemolysis

**Figure 1:**
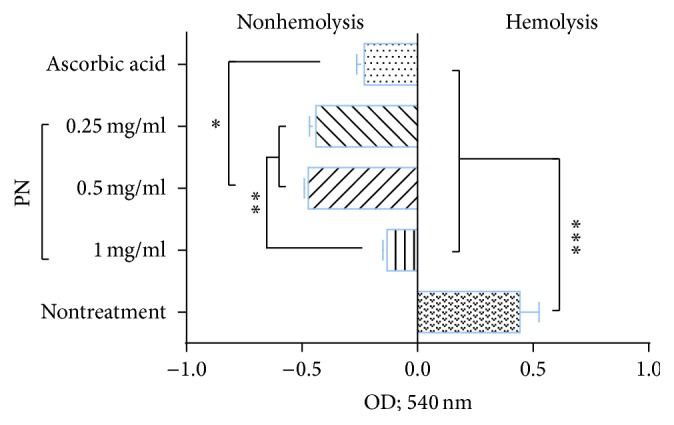
The severity of hemolysis among three doses of PN, ascorbic acid, and nontreatment groups. ^*∗*^ *p* < 0.05; ^*∗∗*^ *p* < 0.01; ^*∗∗∗*^ *p* < 0.001.

Our results revealed that, after induction of oxidative stress to erythrocyte with H_2_O_2_, three doses of PN and ascorbic acid had no evidence of hemolysis compared to nontreatment group (Figure 1). However, the released hemoglobin absorbance in 0.25 and 0.5 mg/mL PN treated groups was significantly lowered compared to other groups. These indicated that all doses of PN and ascorbic acid can protect hemolysis from H_2_O_2_-induced oxidative stress on intact sheep erythrocyte. Like other studies, herbal plants and plant-derived antioxidant, such as flavonoids, orientin, and luteolin, have been used to alleviate oxidative stress on erythrocyte [16, 22, 23]. PN also has antioxidative effect to protect hemolysis corresponded to our previous studies that claim an antioxidative property of PN on endothelial stress and oxidative stress induced by apoptosis [13]. Inhibitory effect of PN on cellular apoptosis is relevant to the integrity of red blood cell. Moreover, the main active ingredients of PN in association with antihemolytic property need to be clarified by further studies because it composes several kinds of Thai herbal plants. However, the possible compounds from each herb in PN regarding the preventive role in hemolysis are antioxidants as mentioned by the previous studies.

### 3.2. Dysmorphic Erythrocyte

**Figure 2:**
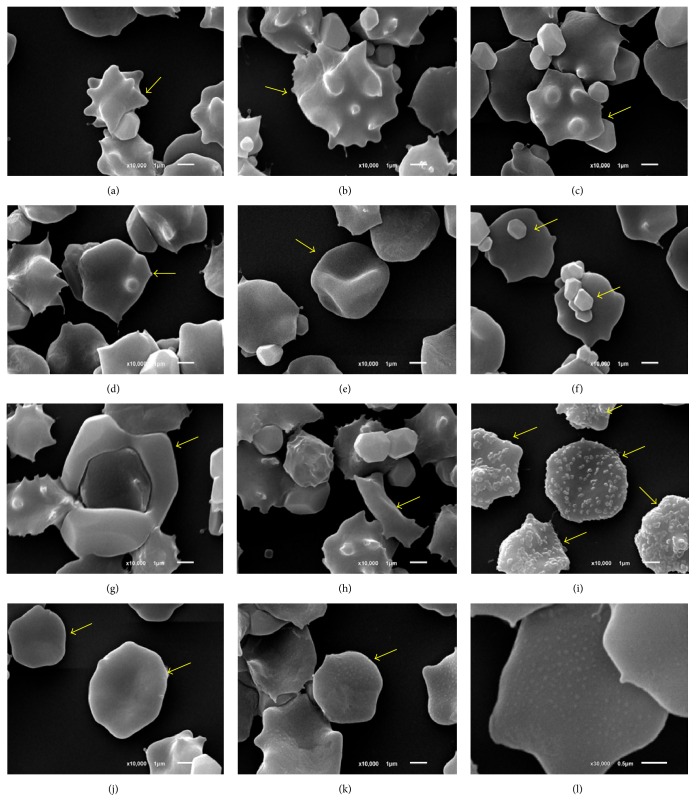
Scanning electron micrograph of dysmorphic erythrocyte induced by oxidative stress. (a-b) Echinocyte, (c) acanthocyte, (d) small particle on erythrocyte, (e) knizocyte, (f) erythrocyte clumping, (g) codocyte, (h) bizarre shaped erythrocyte, (i) irregular membrane, (j) intact erythrocyte with a bit biconcave, (k) rough membrane, and (l) higher magnification of rough membrane.

As a consequence to the oxidative stress reaction induced by H_2_O_2_ in intact sheep erythrocyte, there were several kinds of dysmorphic erythrocyte, as shown in Figure 2. Echinocyte was a spiny erythrocyte with various stages of blunt or sharp surface projection called erythrocyte crenation (Figures 2(a) and 2(b)). Acanthocyte formed several knobs on erythrocytic membrane (Figure 2(c)). Knizocyte was triconcave erythrocyte or called pinched cell characterized by the central bar of hemoglobin beside clear space areas on both sides (Figure 2(d)). Codocyte formed a single knob on erythrocytic membrane called target cell (Figure 2(e)). Some of erythrocyte abnormalities such as small particle on erythrocytic surface (Figure 2(f)), erythrocyte clumping (Figure 2(g)), bizarre shaped (Figure 2(h)), irregular membrane (Figure 2(i)), and rough membrane (Figures 2(k) and 2(l)) were found.

**Figure 3:**
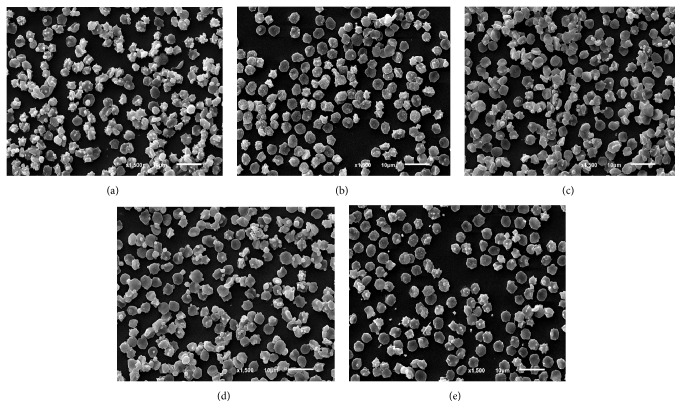
Scanning electron micrograph of oxidative erythrocyte among (a) nontreatment, (b) ascorbic acid, and (c–e) all doses of PN (0.25, 0.5, and 1.0 mg/mL).

The distribution of dysmorphic erythrocyte among groups was showed in Table 1 and Figure 3. The number of normal erythrocytes in ascorbic acid and all doses of PN was significantly higher than nontreatment group. Mild defect erythrocytes such as rough and irregular membranes in few doses of PN and ascorbic acid were also higher number than nontreatment group. The number of echinocytes and knizocytes was significantly found in nontreatment group, while acanthocyte was significantly found in ascorbic acid and 1 mg/mL PN. In addition, there was no difference in number of the remaining dysmorphic erythrocytes among groups of the study.

In general, the variation in shape and size of erythrocyte is called poikilocytosis that occurs due to either membrane abnormalities or trauma. Echinocyte, acanthocyte, and codocyte are erythrocytic membrane defect. Echinocyte formation has been described in H_2_O_2_-induced oxidative stress on red blood cells [24, 25]. In our study, H_2_O_2_-induced oxidative stress caused a number of echinocytes predominately and knizocytes subordinately. Small particle on erythrocyte, erythrocyte clumping, codocyte, and bizarre erythrocyte were also minorly found. Echinocyte formation is postulated against hemolysis by increasing plasma membrane surface area relative to cellular volume [26]. It can take up more water as compensation for a given amount of osmotic stress. Recently there is an evidence describing that echinocyte formation in H_2_O_2_-induced oxidative stress is caused by SO_4_^2−^ transported defect [24]. Therefore, cellular volume of echinocyte may increase to the highest degree before hemolysis [26]. In addition, erythrocyte under oxidative environment is dramatically changed as follows: (i) hemoglobin alteration, (ii) globin binding to erythrocyte cytoskeleton [27] probably characterized by knizocyte and codocyte, respectively, and then (iii) membrane alteration with mild to severe defects. Following to our results, the possibly cascade events of H_2_O_2_-induced oxidative stress to erythrocyte are rough and irregular membrane, knizocyte (hemoglobin bar), codocyte (single knob), acanthocyte (several knob), echinocyte (crenation), and finally hemolysis. Obviously, PN and ascorbic acid reduced echinocyte and knizocyte formation from H_2_O_2_-induced dysmorphic erythrocyte. The severity of membrane alteration in PN and ascorbic acid was limited to rough and irregular membranes. Acanthocyte was not developed to high number of echinocytes. However, in ascorbic acid and 1.0 mg/mL of PN exhibited high number of irregular membrane when compared to the other groups (Figure 3). These results demonstrated that PN could preserve erythrocyte from oxidative stress in both shape and membrane integrity with being the most effective at 0.5 mg/mL dosage. In addition, the incidence of dysmorphic erythrocyte varies and depends on disease stages. Similar to the present study, metabolic syndrome-induced oxidative stress increases the number of acanthocytes, stomatocytes, and echinocytes when compared to healthy subjects [28].

### 3.3. AQP-1 Expression

**Figure 4:**
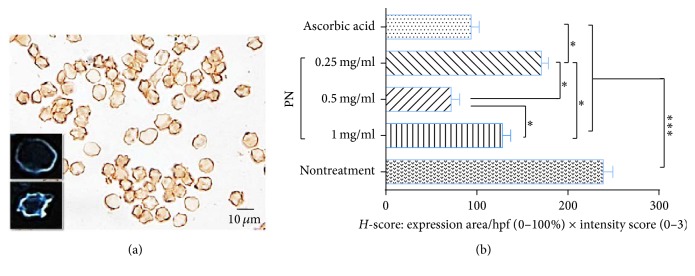
AQP-1 expression on erythrocyte surface. (a) AQP-1 immunohistochemical staining with DAB; inset indicated the adjusted images of intact (upper) and dysmorphic (lower) erythrocyte and (b) *H*-score of AQP-1 expression among three doses of PN, ascorbic acid, and nontreatment groups. ^*∗*^ *p* < 0.05 and ^*∗∗∗*^ *p* < 0.001.

To determine the alteration of osmotic ability on sheep erythrocytic membrane after H_2_O_2_-induced oxidative stress, immunohistochemical staining of AQP-1 was conducted. The results revealed that AQP-1 was expressed on erythrocytic membrane with difference level of expression between intact and defective erythrocytes. Defected erythrocyte (Figure 4(a), lower inset) had higher AQP-1 expression than the intact (Figure 4(a), upper inset). *H*-score of AQP-1 expression indicated that rank of expression from the highest to lowest was nontreatment, 0.25 mg/mL PN, 1.0 mg/mL PN, ascorbic acid, and 0.5 mg/mL PN (Figure 4(b)) at significance level.

Erythrocytes are anucleated cells with hemoglobin storage that allow oxygen to be transported in the circulation throughout of body. Several cardiovascular diseases, particularly coronary artery disease and hypertension, have been claimed that they are caused by oxidative stress-induced defect on erythrocytic membrane integrity and fluidity [7, 29–31]. Erythrocytic membrane is composed of a typical lipid bilayer and plays many important roles in association with erythrocyte surface deformity, permeability, fluidity, and flexibility via some membrane protein channels especially AQP-1, erythrocytic water transporter membrane protein [32]. An alteration of AQP-1 expression on erythrocyte is related to its membrane integrity as proven by several reports; water intoxication produces upregulation of AQP-1 on red blood cell [33]; the increment of AQP-1 expression leads to increased water influx to erythrocyte consequence to high osmotic pressure as seen in red blood cell ghost [34]; recently, AQPs, both AQP-1 and AQP-8, are efficient transmembrane diffusion of H_2_O_2_; therefore, H_2_O_2_ can promote permeability of AQPs [35, 36]. In the present study, the results indicated that dysmorphic erythrocyte induced by H_2_O_2_ had increased AQP-1 expression. In relation to AQP-1 expression, this referred to the fact that osmotic ability was increased in dysmorphic erythrocyte, especially echinocyte and knizocyte, before reaching the greatest cellular volume and developing to hemolysis. All doses of PN and ascorbic acid lowered AQP-1 expression when compared to nontreatment group. Therefore, PN had ability to preserve and maintain osmotic ability on erythrocyte to protect hemolysis particularly at 0.5 mg/mL dosage similar to ascorbic acid. However, the protective mechanism of PN on echinocyte formation in association with H_2_O_2_-induced upregulation of AQP-1 is required for further studies.

## 4. Conclusions

PN ameliorated dysmorphic erythrocyte induced by oxidative stress. It also decreased hydrostatic pressure on erythrocyte in relation to the reduction of AQP-1 expression. Antioxidative property of PN may preserve erythrocyte morphology in terms of both integrity and osmotic ability and could be a useful herbal medical product to alleviate some kinds of cardiovascular symptoms.

## Figures and Tables

**Table 1:** Distribution of dysmorphic erythrocyte among three doses of PN, H_2_O_2_, and ascorbic acid.

Erythrocyte morphology (%)	H_2_O_2_ (nontreatment)	Ascorbic acid	Phikud Navakot (mg/mL)		
			1	0.5	0.25
Normal RBCs	0	19.5 ± 5.4^*∗∗*^	20.5 ± 9.5^*∗∗*^	25.2 ± 8.2^*∗∗*^	20.6 ± 11.2^*∗∗*^
Normal RBCs with rough membrane	0.7 ± 0.03	0	0	70.3 ± 24.1^*∗∗∗*^	50.1 ± 12.3^*∗∗∗*^
Irregular membrane	0	48.3 ± 11.8^*∗∗∗*^	50.3 ± 6.4^*∗∗∗*^	0	10.8 ± 12.3^*∗*^
Echinocyte	70.4 ± 5.9	0.4 ± 0.02^*∗∗∗*^	0.4 ± 0.01^*∗∗∗*^	0.6 ± 0.08^*∗∗∗*^	5.1 ± 2.9^*∗∗∗*^
Small particle on RBCs	20.3 ± 8.3	9.2 ± 2.3	10.8 ± 1.1	5.6 ± 1.1	5.2 ± 0.3
Acanthocyte	5.6 ± 2.3	21.4 ± 8.4^*∗*^	20.3 ± 4.3^*∗*^	0	5.4 ± 3.1
Knizocyte	25.4 ± 8.1	0^*∗∗∗*^	0^*∗∗∗*^	0^*∗∗∗*^	0^*∗∗∗*^
RBC clumping	2.0 ± 1.1	0	0	0	0
Codocyte	0	0	0	0	5.0 ± 2.1
Bizarre shaped	0.5 ± 0.01	0	0	0	0

^*∗*^Significantly different to H_2_O_2_, ^*∗*^ *p* < 0.05, ^*∗∗*^ *p* < 0.01, and ^*∗∗∗*^ *p* < 0.001.
